# Three Cases of Eye, Nasal and Throat Trouble—Illustrative of the Importance of Recognizing Syphilis as a Cause in Such Cases

**Published:** 1886-04

**Authors:** R. O. Cotter

**Affiliations:** Macon, Ga.


### THREE CASES OF EYE, NASAL AND THROAT TROUBLE—ILLUSTRATIVE OF THE IMPORTANCE OF RECOGNIZING SYPHILIS AS A CAUSE IN SUCH CASES.

By R. O. COTTER, M. D., Macon, Ga.

*Case 1.*—A gentleman, aged 30, consulted me on November 23 for a trouble which had begun two months previously. He had, during this time, passed through the hands of four physicians of unquestioned ability in this city. When I first saw him there was a peculiar looking, ulcerated abscess half inch below the inner canthus of the right eye on the side of the nose corresponding to the lower edge of the lachrymal sac. He also had a stricture of the nasal duct and a constant overflow of tears, though this latter was a recent trouble. At a first glance I took the abscess to be what we very frequently find in this locality, an ulcerated sebaceous cyst. The fistula just barely communicated with the lachrymal sac. As there was no purulent matter issuing from the canaliculus on pressure, I soon saw that it was not a common chronic case of stricture with fistulous abscess, but was disposed to think the fistula might have been caused by an unsuccessful though not improper attempt of one of his former physicians who had attempted to destroy what was supposed to be an ulcerated sebaceous cyst, with a free use of stick of nitrate of silver. Using a 4 per cent. solution of cocaine, I operated upon the stricture of the duct by slitting up the canaliculus in the usual manner with a probe-pointed canaliculus knife, and then cutting the stricture—afterwards introducing at intervals of three to six days up to the present time Nos. 6 and 8 of Bowman's probes. The stricture has given no further trouble, and the overflow of tears has entirely stopped. After waiting several days, the ulcer or fistula showed no signs of healing up, and I began to suspect either syphilis or epithelioma, though he denied most positively that he had ever had syphilis, and as he was a highly intelligent gentleman and did not deny having frequently laid himself liable to such trouble, I accepted his assurance. He did indeed have partially atrophied testicle, but as he had had varicocele for several years, this could account for it. As the fistula did not heal up promptly, as they usually do after the stricture is operated upon, but continued to swell up and discharge a thin sanious pus, I began strongly to suspect epithelioma, or the existence of carious bone, but careful exploration for the latter did not reveal it.

On December 1st, I again laid open the fistula, and after applying a 4 per cent. solution of cocaine, I thoroughly cauterized it with a stick of caustic potash. The cocaine so mitigated the pain as to render it much less than to have done without it. The caustic was applied nearly half inch deep and was very thorough. I checked its further action by applying some dilute acetic acid on a pledget of cotton. I packed the ulcer every day with lint soaked in an ointment of oxide of zinc ointment and vaseline, hoping to get the ulcer to heal up from the bottom. I will state here that no tears now oozed through the fistula. But the ulcer would not heal up from the bottom, though it would readily heal up on the outside. This latter fact tended to fortify against the probability of epithelioma.

The patient still denied having had any venereal trouble.

While I do not believe much in the supposition that iodide of potash will cause atrophy of the testicles, although so high an authority as Prof. Alfred Stillé thinks such an idea is not altogether hypothetical, as my patient informed me that his testicle had begun to resume some of its former size within the past few months, I concluded to give him the benefit of refraining from the use of the iodide.

By the way, I will add, the atrophy of the testicle began seven years ago.

Yet, as the ulcer would not heal up from the bottom, I finally determined to give him the iodide of potash, and on December 22, I began him on 5 gr. three times daily, and gradually increased the dose to 30. gr. The ulcer showed decided improvement in three or four days, and was well by January 3d. On December 29th, he stated to me that, after carefully recalling over events, he recollected having a very small sore come on his penis when he was 19 years old, which his doctor assured him would amount to nothing, though he recollected the doctor treated him for about two weeks and salivated him; so I suppose he took a two-weeks' course of mercury. He finally recollected that "following the small sore he had a sore throat, his hair came out somewhat, and he had some small lumps in his groin."

I know this gentleman well enough to believe that he was perfectly innocent of any intention to deceive me. Yet as to the direct cause of his trouble being syphilis, could we ask for plainer proof?

*Case 2.*—Young man, aged 17, was brought to me on April 22d for treatment of an eye trouble which had existed for nearly two months. He had been treated up to this time by two very experienced general practitioners, but his mother stated that his eyes were growing rapidly worse. An examination showed that he had intense iritis in both eyes with adhesions of the iris to lens. Although at this visit he denied having ever had any venereal trouble, on the following day, when he was sent to the office without his mother, I made a careful examination, and found a scar of a recently-healed chancre on his penis and enlarged lymphatic glands of the groins in addition to the sore throat which I had observed the day before. He was put upon full doses of iodide of potash with inunctions of mercurial ointment a 3-gr. sol. of sulph. atropia was used in the eye, hot water applications to the eye, together with blisters alternately to the temples and behind the ears.

By the 1st of July his eyes were entirely well and his vision normal. I then turned him over to the family physician to treat him for syphilis. The doctor, though a man of undoubted ability, admitted to me that he had not suspected the boy of having any venereal trouble.

*Case 3.*—A man, aged 55, otherwise apparently healthy, consulted me on November 23d for loss of voice, which he thought came on from bathing in cold water during last August. He could not recollect having any inflammatory sore throat, but that, after slight symptoms of sore throat, had gradually lost his voice. He could not speak above a very hoarse whisper, and also had difficulty in swallowing.

Examination with the laryngoscope showed partial paralysis of both vocal cords and of the epiglottis, but no laryngitis of any consequence.

He was put upon 1–32 gr. strychnia and 10 grs. iodide of potash three times daily.

On December 15th he again called at my office greatly improved. At this second examination, he confessed that he had a small sore on his penis seventeen years ago, but had hardly thought it necessary to confess such a small matter to me before. He was ordered to continue the treatment and to double the doses of each medicine.

At a third visit a few days ago, he showed more improvement and could talk very much better and easier.

While I hardly hope for a complete recovery in his case, I certainly look for a very satisfactory improvement.

